# Integrative network analysis reveals USP7 haploinsufficiency inhibits E-protein activity in pediatric T-lineage acute lymphoblastic leukemia (T-ALL)

**DOI:** 10.1038/s41598-021-84647-2

**Published:** 2021-03-04

**Authors:** Timothy I. Shaw, Li Dong, Liqing Tian, Chenxi Qian, Yu Liu, Bensheng Ju, Anthony High, Kanisha Kavdia, Vishwajeeth R. Pagala, Bridget Shaner, Deqing Pei, John Easton, Laura J. Janke, Shaina N. Porter, Xiaotu Ma, Cheng Cheng, Shondra M. Pruett-Miller, John Choi, Jiyang Yu, Junmin Peng, Wei Gu, A. Thomas Look, James R. Downing, Jinghui Zhang

**Affiliations:** 1 Department of Computational Biology, St Jude Children’s Research Hospital, 262 Danny Thomas Place, MS321, Memphis, TN 38105 USA; 2 Center for Proteomics and Metabolomics, St Jude Children’s Research Hospital, Memphis, USA; 3 Department of Biostatistics, St Jude Children’s Research Hospital, Memphis, USA; 4 Department of Pathology, St Jude Children’s Research Hospital, Memphis, USA; 5 Department of Cell and Molecular Biology, St Jude Children’s Research Hospital, Memphis, USA; 6 Departments of Structural Biology and Developmental Neurobiology, St Jude Children’s Research Hospital, Memphis, USA; 7 Department of Pathology and Cell Biology and Institute for Cancer Genetics, Columbia University, New York, USA; 8 Department of Pediatric Oncology, Dana-Farber Cancer Institute, Harvard Medical School, Boston, MA 02216 USA

**Keywords:** Acute lymphocytic leukaemia, Gene regulatory networks, Computational biology and bioinformatics

## Abstract

*USP7,* which encodes a deubiquitylating enzyme, is among the most frequently mutated genes in pediatric T-ALL, with somatic heterozygous loss-of-function mutations (haploinsufficiency) predominantly affecting the subgroup that has aberrant *TAL1* oncogene activation. Network analysis of > 200 T-ALL transcriptomes linked *USP7* haploinsufficiency with decreased activities of E-proteins. E-proteins are also negatively regulated by TAL1, leading to concerted down-regulation of E-protein target genes involved in T-cell development. In T-ALL cell lines, we showed the physical interaction of USP7 with E-proteins and TAL1 by mass spectrometry and ChIP-seq. Haploinsufficient but not complete CRISPR knock-out of *USP7* showed accelerated cell growth and validated transcriptional down-regulation of E-protein targets. Our study unveiled the synergistic effect of *USP7* haploinsufficiency with aberrant *TAL1* activation on T-ALL, implicating *USP7* as a haploinsufficient tumor suppressor in T-ALL. Our findings caution against a universal oncogene designation for *USP7* while emphasizing the dosage-dependent consequences of USP7 inhibitors currently under development as potential cancer therapeutics.

## Introduction

T-cell acute lymphoblastic leukemia (T-ALL) accounts for 15% of newly diagnosed patients with pediatric acute lymphoblastic leukemia (ALL)^1,2^. Subgroups of T-ALL are defined by aberrant expression of oncogenic transcription factors activated by somatically acquired structural re-arrangements or non-coding regulatory variants^3^. These oncogenic transcription factors include basic helix–loop–helix (bHLH) transcription factors, such as *TAL1*, *TAL2*, and *LYL1*; LIM-only domain genes, such as *LMO1* and *LMO2*; and other transcription factors, such as *HOXA*, *TLX1*, and *TLX3*^4^. They re-wire the regulatory network for T-cell development^5^, thus disrupting the normal regulatory mechanisms controlling cell growth and survival^4,6^.

Our previous study on the genomic landscape of pediatric T-ALL identified ubiquitin-specific peptidase 7 (*USP7*) as one of the most frequently mutated genes^5,7^. USP7 is a deubiquitinating enzyme (DUB) that removes ubiquitin attached by E3 ubiquitin ligases^8^ and is implicated in diverse processes regulating human cell biology and physiology^9^. Although de novo *USP7* germline mutations have been found to cause neurological disorders by disrupting its regulation of MAGE-L2-TRIM27^10^, USP7 is best known to regulate the MDM2-TP53 axis via deubiquitination^11^, affecting several downstream pathophysiological processes such as DNA repair, immune response, and cancer. Other cancer driver genes regulated by *USP7* include *RB1* in glioma^12^, *WNT* in colorectal cancer^13^, *MYCN* in neuroblastoma^14^, *PTEN* in promyelocytic leukemia^15^, and *NOTCH1* in T-ALL^16^.

**Figure 1:**
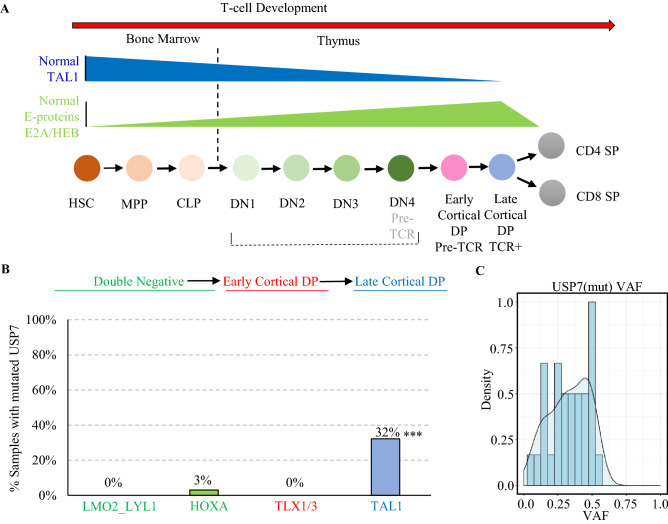
*USP7* haploinsufficiency is enriched in the TAL1-activated T-ALL. (**A**) TAL1 and E-protein activities at different stages of normal hematopoiesis adapted from Sanda and Leong^55^. TAL1 is active during the early stage of T-cell development but down-regulated throughout T-cell maturation. E-protein, which is negatively regulated by TAL1, shows an opposite pattern. The blue color shows the decreasing TAL1 activity in normal hematopoiesis development. The green color shows E-protein activity in normal hematopoiesis development. (**B**) Prevalence of USP7 mutations in the four major T-ALL subgroups with ≥ 5% incidence in the TARGET T-ALL cohort. All but one of the USP7 mutations are in the TAL1 subgroup, and the enrichment is statistically significant (***p-value = 1.1e−11 by a two-sided Fishers-exact test). Representative T-ALL subgroups are ordered by their T-cell development state. (**C**) Density plot of the variant allele frequency (VAF) distribution of USP7 somatic mutations.

Currently, *USP7* is considered an oncogene and a therapeutic target based on its pattern of overexpression and CRISPR screening in several cancers^17–20^, and many USP7 inhibitors have been developed^16,17,21–23^. However, the presence of somatic loss of function (LOF) mutations in T-ALL suggests that *USP7* may function as a tumor suppressor gene in T-ALL. Intriguingly, *USP7* mutations occur almost exclusively in the *TAL1* T-ALL, a subgroup that has aberrantly activated expression of the *TAL1* oncogene. In normal thymocyte development, *TAL1* is expressed in the early stages, such as hematopoietic stem cells (HSCs) and double-negative immigrant thymocytes. E2A and HEB, both E-proteins of class I bHLH transcription factors, become expressed as the thymocyte matures and act as inhibitors of early thymocyte proliferation^24,25^ (Fig. 1A). In T-ALL, aberrantly activated TAL1 is dependent on E2A and HEB for malignant transformation, because as a class II bHLH, TAL1 must heterodimerize with E2A or HEB in order to bind E boxes in its target enhancers^26^. The high prevalence of *USP7* haploinsufficiency in *TAL1* T-ALL suggests that *USP7* may be an important player in the TAL1/E-protein regulatory network.

To investigate this, we combined patient sample analysis with experiments in models of childhood T-ALL, which involves analyzing (1) the transcriptional regulatory network underlying primary T-ALL using RNA-seq data generated from 206 patient samples; and (2) transcriptomic and phenotypic changes caused by *USP7* knock-out in T-ALL cell lines. The results consistently showed that *USP7* haploinsufficiency led to a significant decrease in the E-protein activity of E2A and HEB. Physical co-localization of USP7 with members of the E-proteins and TAL1 was validated by affinity purification followed by mass spectrometry (AP-MS) as well as by re-analysis of publicly available chromatin immunoprecipitation with DNA sequencing (ChIP-seq) data of T-ALL cell lines. Moreover, the *USP7* partial (50%) knock-down in a *TAL1* T-ALL cell line uniquely increased cell growth. Our study unveils important biological processes affected by *USP7* haploinsufficiency, demonstrating the dosage-dependent molecular pathogenesis of *USP7* loss in T-ALL development.

## Methods

### Cell culture, transfections, transductions, shRNAs, and CRISPR/Cas9

HEK-293T cells (ATCC) were cultured in DMEM-10% FBS medium. Human T-ALL cell lines Jurkat (ATCC), HSB-2 (ATCC), and Molt-4 (ATCC) were cultured in RPMI1640-10% FBS medium. USP7 shRNAs lentivirus was generated by co-transfecting 293T cells with shRNA vectors (OriGene or Sigma) (shRNA sequences listed in the Supplementary Table S5), and packaging plasmids. T-ALL cells were transduced with USP7 shRNA lentivirus and sorted for GFP positive cells five days after transduction or selected by puromycin. The knock-down of USP7 by shRNA was validated by real-time PCR and western blot. Genetically modified T-ALL cells were generated using CRISPR-Cas9 technology. Editing construct sequences and screening primers are listed in Supplementary Table S11.

### CRISPR/Cas9 construction

Briefly, the sgRNA was designed with at least 3 bp of mismatch to any other site in the human genome to mitigate the risk of off-target editing. To generate the modified cells, 400,000 cells were transiently co-transfected with/without 0.5 μg pmaxGFP plasmid (Lonza), with 33 pmol spCas9 protein, 100 pmol chemically modified sgRNA (Synthego), and 100 pmol of blocking ssODN (IDT) via nucleofection (Lonza), using solutions and programs according to the manufacturer’s recommended protocol. Five days post-nucleofection, cells were single-cell sorted by fluorescence-activated cell sorting into 96-well plates. Cells were clonally expanded and verified the desired modification via targeted deep sequencing followed by CRIS.py analysis^27^. Selected homo/hetero knock-out clones were validated by MiSEQ, Sanger sequencing, RNAseq as well as western blotting to confirm the expression of USP7. We note that T-ALL cells tend not to tolerate Cas9 that well, which could explain our difficulty in generating viable CRISPR clones in HSB-2 cells.

### Cell growth assay

T-ALL cells were seeded in 96-well plates at the concentration of 10^4^/well. Cell growth titers were determined using the CellTiter-Glo Luminescent Cell Viability Assay kit (Promega). Each assay contained triplicate samples and was performed twice for reproducibility. Luminescence was measured using SpectraMax i3X (Molecular Devices).

### Sample preparation for affinity purification and mass spectrometry

The analysis was performed following our optimized protocol^28^. Gel bands were reduced with dithiothreitol (DTT) (Sigma) and alkylated by iodoacetamide (IAA) (Sigma). The gel bands were then washed, dried, and rehydrated with a buffer containing trypsin (Promega). Samples were digested overnight, acidified, and the peptides were extracted. The extracts were dried and reconstituted in 5% Formic acid. Peptide samples were loaded on a nanoscale capillary reverse phase C18 column by an HPLC system (Thermo EASY-nLC 1000) and eluted by a gradient. The eluted peptides were ionized and detected by an inline mass spectrometer (Thermo LTQ Orbitrap Elite). The MS and MS/MS spectra were collected over an 80-min liquid chromatography gradient. In some instances, the peptide was not detected due to transient interactions^29^ or overlapping retention time and peaks. These issues can reduce the detection sensitivity of the mass spectrometer^30^.

### Western blot analysis

For western blot analysis, cells were lysed in RIPA lysis buffer (Thermo Scientific) supplemented with protease/phosphatase inhibitor cocktail (Thermo Scientific). Cell numbers based on an equal amount of cell lysates were loaded and separated in NuPAGE 4–12% Bis–Tris protein gels (invitrogen). Proteins were transferred to PVDF membranes using iBlot (invitrogen) and incubated with indicated antibodies in the iBind system (invitrogen) (antibodies listed in the Supplemental Table S12). The protein bands were detected by SuperSignal West Femto Maximum Sensitivity Substrate (Thermo Scientific).

### Reverse transcription-quantitative PCR

cDAN was generated using the High-Capacity cDNA Reverse Transcription kit (Applied Biosystems, Cat # 4368814). Real-time PCR was performed using PowerUp SYBR Green Master Mix (Applied Biosystems, Cat # A25918) on CFX real-time PCR system and data were analyzed using CFX Maestro qPCR Analysis software (BIO-RAD) (Primers are listed in the Supplemental Table S13). Beta-actin was used as a housekeeping gene for normalizing quantitative mRNA expressions. Each group contains a duplicate batch and a quadruplicate batch to separately validate the results. We note a lack of RAG1 and PTCRA expression in HSB-2 cells.

### Immunoprecipitation

Cell lysates were derived from the IP lysis buffer (Thermo Scientific). The cell lysates were pre-cleaned with mouse IgG and then incubated with agarose beads conjugated antibodies overnight in the cold room with consistent shaking. Beads-bound proteins were recovered by boiling in 60 μl of 2× NuPAGE LDS sample buffer for 5 min. 15 μl of recovered proteins were subjected for western blotting with antibodies indicated in the text. Antibodies used for immunoprecipitation in this study include anti-USP7, TAL1, and E2A. Subsequent ubiquitin and TAL1 western blotting were performed following the E2A pull-down (see Supplemental Table S12 for more details).

### RNA-seq sample preparation

RNA samples were isolated using the RNeasy Mini Kit (QIAGEN) and subjected to RNA-seq sequencing. The total stranded RNA library was prepared using the KAPA RNA HyperPrep Kit with RiboErase (HMR) (Roche Sequencing). RNA sequencing was performed on NovaSeq 6000 and Illumina HiSeq 4000 systems (Illumina). Samples subjected to RNA sequencing were listed in Supplemental Table S10. RNAseq data have been deposited in GEO under accession GSE148522 and GSE148523.

### RNA-sequencing analysis

RNA-seq reads were mapped by our in-house mapping pipeline STRONGARM developed for the Pediatric Cancer Genome Project (PCGP)^31^. Each read pair was aligned to four different databases using BWA, and the best alignment was selected as the final mapping location: (i) the GRCh37-lite reference sequence for human samples (ii) RefSeq; (iii) a sequence file representing all possible combinations of non-sequential pairs in RefSeq exons; and (iv) the AceView database flat file downloaded from the UCSC Genome Browser, representing transcripts constructed from human ESTs. Read count for each gene was obtained with HT-seq^32^. Reads are normalized to fragments per kilobase million (FPKM) for each gene.

Early T-cell precursor (ETP) status was defined using the WARD hierarchical clustering algorithm using genes from Coustan-Smith et al.^33^ and was further confirmed by known ETP cases defined by immunohistochemistry (93% cases).

Gene set enrichment analyses were carried out using GSEA with MSigDB^34^. Lowly expressed genes with less than one FPKM were filtered out. The GSEA parameters used were “-metric signal2noise -set_min 4 -permute gene_set”. E-protein targets were defined based on gene expression profiling of E2A, HEB, and TAL1 knock-down from GSE29181. E-protein targets include genes down-regulated after E2A and HEB knock-down (< − 0.5 log2FC threshold) and up-regulated after TAL1 knock-down, which represent genes negatively regulated by TAL1. For Jurkat CRISPR models, controls (N = 9) were compared to USP7-heterozygous-KO (N = 18) Jurkat samples. For Molt-4 WT CRISPR models, controls (N = 4) were compared to 50% KO (N = 8) Molt-4 samples. See Supplementary Table S10 for the detailed RNAseq sample list. See Supplementary Table S9 for the detailed gene-lists.

### NetBID analysis

NetBID calculates the transcription factor activity based on their downstream target’s normalized expression. In this study, we applied the NetBID algorithm^35^ (https://github.com/jyyulab/NetBID) to identify “hidden” drivers for T-ALL. First, we utilized SJARACNe with default parameters^36^ to reverse-engineer a T-cell development-specific interactome from a publicly available RNA-seq dataset (GSE93107) containing eight different T-cell differentiation stages, against 1113 transcription factors (TF). Network construction was limited to modulating genes only. To ensure that most modulators were included, we filtered out lowly expressed genes—genes expressed in < 10% of the samples. The interquartile range (IQR) was used to define and exclude unchanged non-modulators. Networks derived from SJARACNe resulted in 14,374 nodes and 366,527 edges. Based on these networks, we calculated the activity scores of eight TFs that interacted with TAL1 based on the STRING database^37^ (default 0.4 medium confidence score) for 206 RNAseq of primary T-ALL samples from TARGET using non-weighted mean. Representative T-ALL subgroups were selected based on prevalence > 5%. Differential activity analysis was performed using the ‘limma’ software^38^.

### ChIP-seq data and analysis

ChIP-seq for E2A, HEB, TAL1, and input in Jurkat cells were downloaded from GSE29180. USP7 ChIP-seq and input DNA from untreated Jurkat cells were downloaded from GSE97435. BWA (version 0.7.12, default parameter) was used to align the reads to the human genome hg19 (GRCh37). Picard (version 2.9.4) was used to mark duplicated reads. Non-duplicated reads were retained by samtools (parameter ‘-q 1 -F 1024’ version 0.1.17). Mapped reads were inspected using the cross-correlation plot generated by SPP^39^ to determine the fragment size for extending the read for each transcription factor. Reads from different sequencing runs or replicates were merged together, and peaks called by MACS2 (version 2.0.9)^40^ using the default cutoff (q = 0.05). Bigwig files were generated with the total number of mapped reads normalized to 15 million. Deeptools^41^ was used to plot heatmaps. The code for ChIP-seq analysis was obtained from Yang et al.^42^.

### Mass spectrometry database search and analysis

Database searches were performed using the Sequest^43^ search engine. All matched MS/MS spectra were filtered by mass accuracy and matching scores to reduce protein false discovery rate to < 1% based on the target-decoy strategy^44^. Spectral counts, matching to individual proteins, reflect their relative abundance in one sample after the protein size is normalized. Label-free quantitative comparison based on spectral counting was used to differentiate the protein interactome from non-specific IgG interactome background control. G-test was used to determine the statistical significance of the differences^45^.

## Results

### *USP7* haploinsufficiency deregulates T-cell developmental gene expression in T-ALL

Our previous study on the genomic landscape of pediatric T-ALL showed that *USP7* is one of the most frequently mutated genes, with an overall 12% mutational prevalence^5^. *USP7* mutations occurred almost exclusively in the *TAL1* subgroup, which has aberrantly activated *TAL1* expression accompanied by developmental arrest at the late cortical double-positive (DP) stage of thymocyte development (Fig. 1B). All mutations are heterozygous (Fig. 1C) and predominantly (79%) loss-of-function truncation variants (Supplementary Table S1). These data suggest that USP7 haploinsufficiency may deregulate T-cell development by synergizing with aberrant *TAL1* activation in T-ALL.

**Figure 2:**
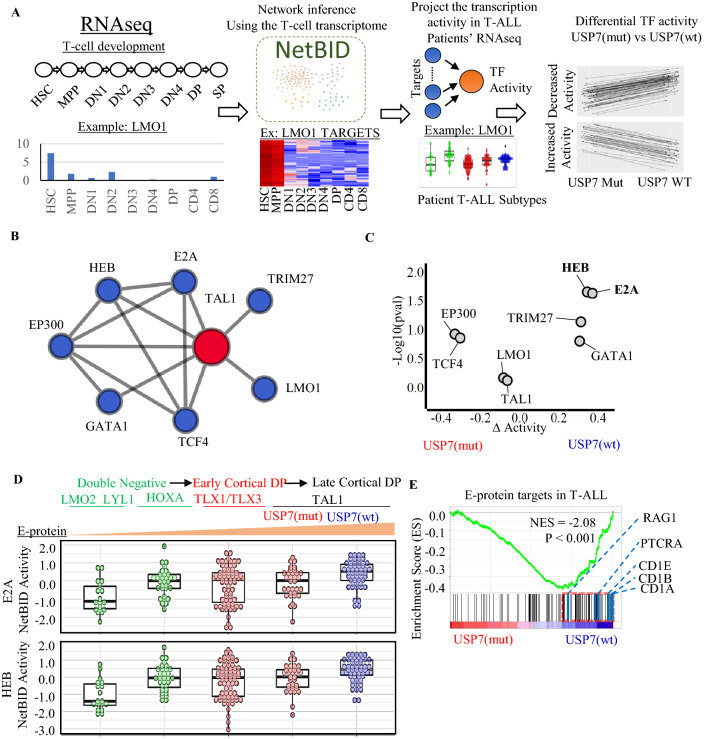
*USP7* haploinsufficiency reduces E-protein activity in NCI TARGET T-ALL cohort. (**A**) NetBID network analysis overview. A transcription factor network was generated based on the transcriptome profiling of T-cell development. Transcription factor activity in T-ALL is estimated using their down-stream targets with NetBID. Dysregulated transcription factors are identified for USP7 haploinsufficiency. (**B**) NetBID identified transcription factors that interact with TAL1. The edges represent known physical interaction from STRINGdb. (**C**) Differential activities of TAL1-interacting transcription factors (TF) based on NetBID analysis. The x-axis presents the change in TF activity between USP7^wt^ and USP7^mut^ *TAL1* T-ALL samples, and the y-axis presents the p-values (− log10) by an unpaired two-sided *t* test. (**D**) E2A and HEB activities across the major T-ALL subgroups. (**E**) Enrichment of E-protein targets in USP7^wt^ compared to USP7^mut^ by GSEA analysis. Leading-edge genes are marked by a dotted red rectangle, and the list on the right shows top hit genes.

To test this hypothesis, we evaluated the predicted activities of TAL1-interacting transcription factors (TF) in 206 T-ALL transcriptomes (RNA-seq) profiled by NCI TARGET^5^. The network analysis was performed using NetBID (Fig. 2A), an algorithm that calculates transcription factor activity based on the expression levels of their downstream targets^35^. Thirty-five T-ALLs identified as early T-cell precursors (ETP) by gene expression signature analysis^33^ were excluded as they resemble a distinct subset of newly immigrated thymocyte precursors with distinct immature immunophenotypes (Supplementary Figure S1A). To examine the TF activities in the context of T-cell differentiation, we constructed a reference TF regulatory network using RNA-seq data generated from normal T-cell development^35^ (Fig. 2A). Focusing on the 8 TFs known to have physical interaction with TAL1 based on the STRING database^37^ (Fig. 2B), we compared the activity scores between mutated *USP7* (USP7^mut^) vs. wild-type *USP7* (USP7^wt^) samples within the *TAL1* subgroup. E-proteins, E2A and HEB, which are known to be negatively regulated by TAL1, emerged as the top-ranked master regulators that were down-regulated in USP7^mut^ T-ALL (Fig. 2C; Supplementary Table S2).

In normal T-cell development, E-protein expression is attenuated during early thymocyte development and increased during the later stages of development, consistent with our analysis of normal T-cell RNA-seq data (Supplementary Figure S1B). Across the T-ALL subgroups, the predicted E-protein activity scores match well with the corresponding T-cell development stages, with attenuated activities in the subgroups associated with early development DN thymocytes (i.e., *LMO2*/*LYL1* and *HOXA*), the gradual increase in the early cortical DP thymocyte subgroup (i.e., *TLX1*/*TLX3* subgroup), and the highest activities in the late cortical DP *TAL1* subgroup (Fig. 2D; Supplementary Table S3). Within the TAL1 subgroup, E-protein activities in USP7^mut^ samples are comparable to those of the early cortical DP thymocyte subgroup. To further evaluate whether *USP7* haploinsufficiency affects E-protein targets in leukemia^46^, we performed a gene set enrichment analysis (GSEA) to assess gene expression changes between USP7^mut^ and USP7^wt^ samples in the *TAL1* subgroup. Genes down-regulated in USP7^mut^ samples were enriched for E-protein targets such as *RAG1* and *PTCRA*^46^ (Fig. 2E), which are targets co-regulated by E-protein (positively) and TAL1 (negatively). Collectively, our analysis suggests that *USP7* haploinsufficiency significantly reduces the expression of E-protein targets in T-ALL.

### *USP7* knock-down in T-ALL cell lines transcriptionally down-regulates E-proteins targets

**Figure 3:**
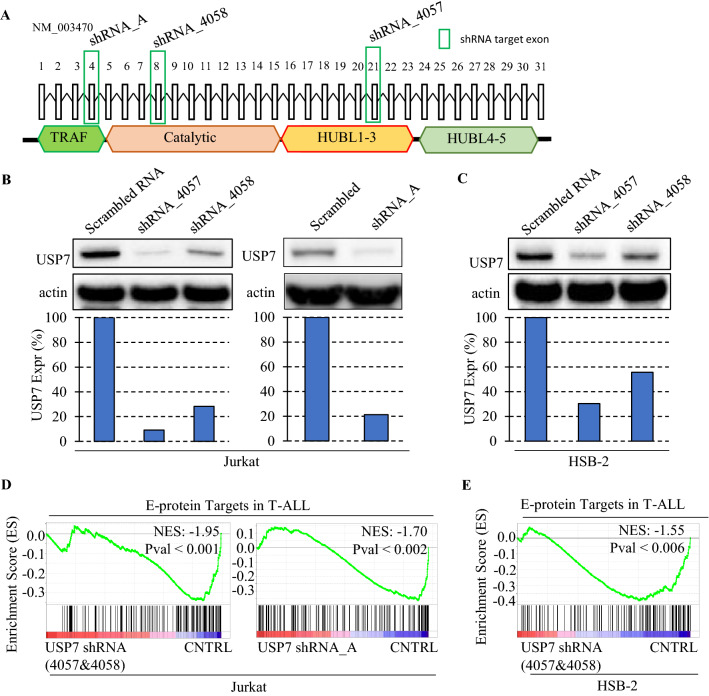
USP7 knock-down in T-ALL cell lines inhibits E-protein targets. Short hairpin RNAs (shRNA) targeting exon 4, 8, and 21 used for knock-down experiments (**A**). Western blot showing the reduction of USP7 protein after shRNA knock-down with the bar plot showing the actin normalized USP7 expression in Jurkat (**B**) and HSB-2 (**C**). GSEA analysis showing shRNA knock-down reduces the expression of E-protein targets compared to the controls in Jurkat (**D**) and HSB-2 (**E**). The number of RNA-seq samples used for this analysis is shown in Supplementary Table S10.

To examine whether the loss of *USP7*, in particular the partial loss of *USP7,* which mimics haploinsufficiency detected in patient T-ALL samples, would reduce transcriptional expression of E-protein targets in T-ALL, we performed a knock-down experiment in T-ALL cell lines. We transduced Jurkat and HSB-2, both T-ALL cell lines with aberrant activation of *TAL1* (i.e., *TAL1* subtype) and intact *USP7*, with lentivirus of *USP7* shRNAs targeting exon 8 (USP7^shRNA_4058^) and exon 21 (USP7^shRNA_4057^), which were used in a previous study^16^, and exon 4 (USP7^shRNA_A^) (Fig. 3A; Supplementary Table S5), which was designed by our study. Western blotting confirmed a partial reduction of *USP7* expression (Fig. 3B,C). To investigate the effect of USP7^shRNA^ on gene transcription, we compared RNA-seq data of Jurkat and HSB-2 cells treated by USP7^shRNA^ versus scrambled control (USP7^wt^). GSEA analysis showed that E-protein targets, which are negatively co-regulated by TAL1, were significantly down-regulated in USP7^shRNA^ Jurkat and HSB-2 cell lines, consistent with the pattern observed in USP7^mut^ T-ALL patient samples (Fig. 3D,E; Supplementary Figure S2A–F). Known E-protein targets such as RAG1 and PTCRA^47^, down-regulated in USP7^mut^ patient samples as well as USP7^shRNA^ Jurkat cells, were validated by RT-qPCR (Supplementary Figure S2G and S2H). Altogether, these results indicated that USP7 consistently regulates E-protein targets in the context of T-ALL with aberrant activation of TAL1, further implicating USP7’s involvement in regulating E2A and HEB activities.

### USP7 forms a co-regulatory complex with TAL1, E2A, and HEB in T-ALL

**Figure 4:**
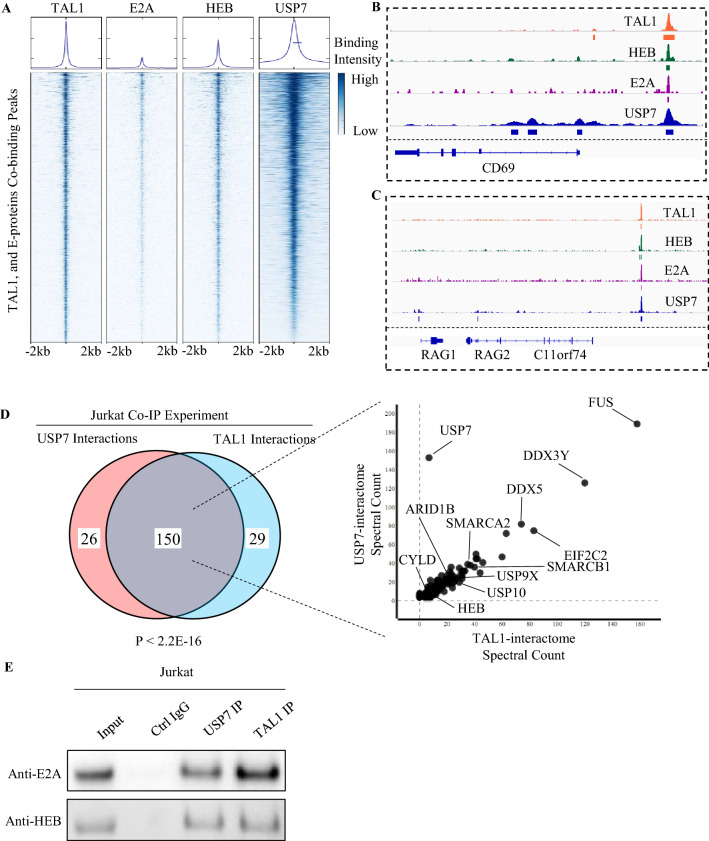
USP7 co-localizes with TAL1, E2A, and HEB in the Jurkat cell line. (**A**) Density heatmap showing that the majority of the peaks from TAL1, E2A, and HEB ChIP-seq overlap with those of USP7. (**B**) Representative TAL1, HEB, E2A, and USP7 peaks proximal to the CD69 gene body. (**C**) Representative TAL1, HEB, E2A, and USP7 peaks proximal to the RAG1/2 gene body. (**D**) Affinity purification-mass spectrometry using USP7 and TAL1 as bait proteins. Venn diagram (left) shows the overlap of USP7 and TAL1 interacting proteins. P-value was calculated based on a two-sided Fishers-exact test. Spectral counts of USP7 and TAL1 interacting proteins are shown on the right. (**E**) Serial co-IP Western blotting showing the interaction of USP7/TAL1 and E proteins.

Down-regulation of E2A and HEB targets by USP7 haploinsufficiency, evident in the analysis of patient T-ALL samples and shRNA knock-down in T-ALL cell lines, prompted us to evaluate whether USP7 forms a co-regulatory complex with TAL1, E2A, and HEB. We first examined the genome-wide co-occupancy of USP7 with these transcription factors by analyzing publicly available ChIP-seq data of Jurkat cells. We found that the majority of ChIP-seq peaks of TAL1 (70.97%), E2A (91.53%), and HEB (79.20%) overlap with USP7 ChIP-seq peaks (p < 2.2E−22 by a two-sided Fishers-exact test; Fig. 4A), demonstrating co-localization of USP7 across the genome with these sequence-specific DNA binding transcription factors. As an example, the co-localization of TAL1, E2A, HEB, and USP7 peaks are proximal to *CD69, RAG1,* and *RAG2,* which are important genes involved in thymocyte differentiation and T-cell activation (Fig. 4B,C).

To determine whether USP7 directly interacts with the TAL1/E-protein complex, we performed an affinity purification-mass spectrometry experiment in the Jurkat cell line using USP7 and TAL1 as bait proteins. We identified 176 proteins interacting with USP7 (Supplementary Table S6), and 179 proteins interacting with TAL1 (Supplementary Table S7). By overlapping the USP7 interactome with the TAL1 interactome, eighty-five percent of the TAL1 protein-partners were shared with the USP7 interactome (Fig. 4D; Supplementary Table S8; p < 2.2E−16). Serial co-IP Western blotting in the Jurkat cell line showed that E2A and HEB physically interact with both USP7 and TAL1 (Fig. 4E). Overall, our results further support USP7’s involvement in regulating the E-protein and TAL1 protein complex.

### USP7 haploinsufficiency promotes T-ALL cellular growth

**Figure 5:**
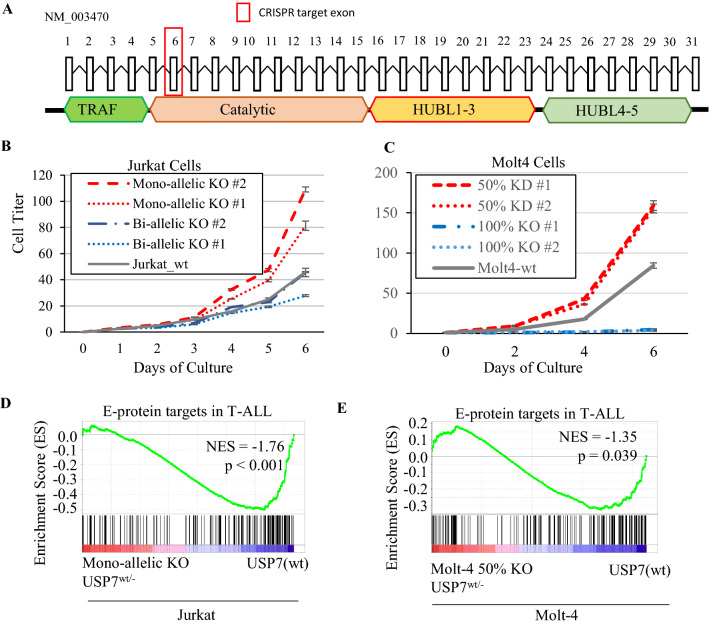
*USP7* haploinsufficiency uniquely promotes cellular growth in Jurkat and Molt-4 cell lines. (**A**) *USP7* haploinsufficiency generated by CRISPR genome editing targeting USP7’s exon 6. (**B**) ATP monitoring system cellular growth assay for the USP7 homozygous KO, USP7 heterozygous KO, and USP7 wild-type control in Jurkat cells. (**C**) Cellular growth assay of the wild-type and CRISPR clones in Molt-4. (**D**) GSEA analysis showing that genes down-regulated in the USP7-heterozygous-KO Jurkat cells compared to USP7-wt control are enriched for E-protein targets negatively regulated by TAL1. (**E**) GSEA analysis showing that genes down-regulated in the USP7 50% KD Molt-4 cells compared to wild-type are enriched for E-protein targets negatively regulated by TAL1.

The uniformity of heterozygous mutations in T-ALL patient samples indicates a potential dosage effect of pathogenicity caused by *USP7* loss in T-ALL. To evaluate this possibility, we used the CRISPR-Cas9 technology targeting exon 6 of *USP7* in Jurkat and Molt-4, the T-ALL cell lines that have aberrant *TAL1* activation and intact USP7 (Fig. 5A). We generated four unique clones in Jurkat (Supplementary Figure S3A) and Molt-4 (Supplementary Figure S4A), respectively; two of which had 50% *USP7* knock-out (KO) mimicking the haploinsufficiency observed in patient samples, and the other had 100% *USP7* KO. In clones with 50% allelic knock-out, we observed reduced *USP7* transcription (Supplementary Figure S3B, S4B) with a significant reduction of the mutant allele expression (mutant allele fraction in RNA-seq ranging 12–14%, p < 1E−5 by a two-sided binomial test) (Supplementary Figure S3C, S4C), consistent with nonsense-mediated decay (NMR) expected from the out-of-frame KO mutation. We performed Western blotting to quantify protein abundance of USP7 and TRIM27, a known direct target stabilized by USP7^10,48^. In Molt-4, both USP7 and TRIM27 protein levels decreased in proportion to 50% and 100% *USP7* CRISPR KO (Supplementary Figure S4D). In Jurkat, bi-allelic knock-out cells lacked USP7 protein expression accompanied by reduction of TRIM27 abundance as expected; mono-allelic knock-out cells did exhibit reduction in TRIM27, but unexpectedly, USP7 protein itself did not change (Supplementary Figure S3D), suggesting decreased USP7 activity despite no reduction in USP7 protein quantity (Supplementary Figure S3D, S4D). We also carried out the same CRISPR-Cas9 KO experiment targeting exon 6 in the HSB-2 cell line. However, no HSB-2 clones survived with *USP7* KO.

We performed an ATP monitoring system cell viability assay and found that both Jurkat and Molt4 with 50% *USP7* knock-out induced an increase in cell growth phenotype compared to the wild-type control (Figs. 5B,C). By contrast, clones of complete *USP7* knock-out exhibited a markedly lower cell growth rate compared to the wild-type (Fig. 5B,C). RNAseq profiling showed significant down-regulation of E-protein targets negatively co-regulated by TAL1 in the 50% USP7 knock-out (Fig. 5D,E). RT-qPCR confirmed RAG1 and PTCRA down-regulation by *USP7* KO in Jurkat (Supplementary Figure S5A and S5C) and Molt-4 cell lines (Supplementary Figure S5B, S5D). Collectively, these results demonstrated that USP7 haploinsufficiency induced growth advantage in the TAL1 subtype of T-ALL accompanied by down-regulation of E-protein targets.

## Discussion

While analyzing the genomic landscape of pediatric T-ALL, we discovered a high frequency of heterozygous LOF mutations of *USP7* in the *TAL1* subgroup^5,7,49^, raising questions regarding the molecular basis for haploinsufficient tumor suppressor activity of *USP7* in this malignancy. In the present study, we began by investigating the impact of *USP7* haploinsufficiency on gene expression and by performing a network analysis of primary T-ALL RNA-seq data from more than 200 patients with this disease. We complemented this systems biology analysis by performing experimental validation, i.e., partially knocking down or knocking out *USP7* in T-ALL cell lines with aberrant overexpression of the *TAL1* oncogene. These experiments showed that *USP7* haploinsufficiency decreases the E-protein activity of E2A and HEB and promotes T-ALL cell growth. Interestingly, impaired thymocyte differentiation^50^ and accelerated leukemia tumorigenesis^51^ were reported in previous studies of E-protein haploinsufficiency using murine models, consistent with decreased E-protein activity observed in T-ALL patient samples and model systems with partial loss of *USP7* investigated in this study.

A previous study using the *TAL1* transgenic mouse model showed that TAL1 heterodimers with E2A or HEB affect the expression of different downstream target genes compared to E2A homodimers or E2A-HEB heterodimers^46^. Our results from several different experimental approaches indicate that USP7 is a critical regulator of E-protein hetero- and homo-dimerization, and thus affecting normal thymocyte development and oncogenesis. First, a re-analysis of ChIP-seq data showed that USP7 co-localizes with multiple members of the basic helix–loop–helix proteins, i.e., TAL1, E2A, and HEB, suggesting a potential role of USP7 in regulating transcription of TAL1 and E-protein targets. Second, AP-MS and western blotting both confirmed that USP7 physically interacts with TAL1 and E-proteins. Third, haploinsufficient *USP7* knock-out in T-ALL cell lines showed a significant reduction of targets mediated by E-protein homodimer. As increased TAL1 oncogenic activity was also evident in the accelerated cell growth observed after CRISPR knock-out of half of the *USP7* loci in T-ALL cell lines, we hypothesize that *USP7* haploinsufficiency in T-ALL down-regulates the ability of this deubiquitylating enzyme to remove ubiquitin from E-proteins, leads to enhanced TAL1 heterodimer formation, which favors TAL1-mediated thymocyte transformation.

Of significant interest, O’Neil et al. previously demonstrated that E-protein haploinsufficiency accelerated leukemia tumorigenesis in a T-ALL murine model with *TAL1* oncogene activity^51^. While our study demonstrates *USP7* haploinsufficiency inhibits E-protein activity in T-ALL cell lines, demonstration of the effect of *USP7* haploinsufficiency on accelerating malignant transformation in vivo will require the development of a *USP7* haploinsufficiency murine model with TAL1 oncogenic activity.

In the Jurkat cell line, we observed an interesting phenomenon that reduction in *USP7* transcription caused by mono-allelic knock-out of *USP7* did not reduce USP7 protein level. Such a discrepancy did not occur in the Molt-4 T-ALL cell line. This suggests a potential feed-back regulation that stabilizes the USP7 protein level in haploinsufficient *USP7* Jurkat cells. It will be interesting to investigate if a similar pattern can be found in other de-ubiquitinating proteins in CRISPR knock-out experiments.

In our study, E2A and HEB protein abundance did not change in Jurkat and Molt-4 cells with partial (50%) USP7 KO (Supplementary Figure S3D, S4D), suggesting that decreased E-protein activity in haploinsufficient *USP7* T-ALL cells is unlikely caused by a decrease in E-protein level through a ubiquitin-dependent proteasome degradation. Indeed, we were able to demonstrate E2A de-ubiquitination by USP7 by overexpression in HEK293T cells (Supplementary Figure S6). Although this preliminary analysis proved biochemically that USP7 leads to E2A de-ubiquitination, an endogenous IP capture designed to quantify the transient and dynamic nature of the ubiquitination process^52,53^ is required for future investigation on ubiquitin modifications that regulate E-protein activity.

There is a burgeoning interest in inhibiting USP7 using small molecules as a potential new cancer therapy. This is largely based on dependency exhibited by many types of cancer on USP7, as revealed by large-scale, genome-wide CRISPR-cas9 knock-out studies, including exquisite dependency of human cell lines from kidney, renal cell carcinoma, skin, and melanoma^20^. In fact, total knock-out of *USP7* also blocks cell growth in T-ALL cells, emphasizing the dosage-dependence of heterozygous *USP7* LOF mutations in pediatric T-ALL. Similarly, a prior study by Li et al.^54^ demonstrated the contrasting effect of mono- versus bi-allelic USP7 loss on the MDM2-TP53 pathway in the HeLa cell, i.e., the partial loss of USP7 leads to destabilization of TP53 while complete ablation leads to activation and stabilization of TP53. Our study illustrates the dosage dependence exhibited by haploinsufficient tumor suppressors like *USP7*, cautioning that partial inhibition of USP7 with small molecules might result in increased tumor cell growth, even though complete inhibition of this deubiquitinase leads to cancer cell death. This situation is not unique to *USP7* but applies broadly to genes that exhibit properties of haploinsufficient tumor suppressors. Haploinsufficient tumor suppressors often exhibit cancer cell lethality when completely inactivated, even though the loss of one allele promotes the growth of the cancer cells. Our study emphasizes the need to carefully evaluate dosage-dependent efficacy when considering inhibitors for cancer treatment of USP7 and other genes that are implicated by mutational analysis as haploinsufficient tumor suppressors in some types of cancer.

## Conclusion

In summary, our comprehensive omics analyses combined with experiments in models of childhood T-ALL consistently showed that *USP7* haploinsufficiency down-regulates E-protein activity, resulting in synergistic activation of TAL1-mediated oncogenic activity. Furthermore, our study illustrates the dosage dependence exhibited by haploinsufficient tumor suppressors like *USP7*, cautioning that while complete inhibition leads to cancer cell death, partial inhibition with small molecules might promote tumor cell growth.

## Supplementary Information

Supplementary Figures.

Supplementary Tables.

## Data Availability

RNAseq data have been deposited in GEO under accession GSE148522 and GSE148523. Proteomics data have been deposited under PRIDE accession PXD023377.

## References

[1] Aifantis I, Raetz E, Buonamici S (2008). Molecular pathogenesis of T-cell leukaemia and lymphoma. Nat. Rev. Immunol..

[2] Hunger SP, Mullighan CG (2015). Acute lymphoblastic leukemia in children. N. Engl. J. Med..

[3] Mansour MR (2014). Oncogene regulation. An oncogenic super-enhancer formed through somatic mutation of a noncoding intergenic element. Science.

[4] Van Vlierberghe P, Ferrando A (2012). The molecular basis of T cell acute lymphoblastic leukemia. J. Clin. Investig..

[5] Liu Y (2017). The genomic landscape of pediatric and young adult T-lineage acute lymphoblastic leukemia. Nat. Genet..

[6] Ferrando AA (2002). Gene expression signatures define novel oncogenic pathways in T cell acute lymphoblastic leukemia. Cancer Cell.

[7] Huether R (2014). The landscape of somatic mutations in epigenetic regulators across 1,000 paediatric cancer genomes. Nat. Commun..

[8] Everett RD (1997). A novel ubiquitin-specific protease is dynamically associated with the PML nuclear domain and binds to a herpesvirus regulatory protein. EMBO J..

[9] Harrigan JA, Jacq X, Martin NM, Jackson SP (2018). Deubiquitylating enzymes and drug discovery: Emerging opportunities. Nat. Rev. Drug Discov..

[10] Hao YH (2015). USP7 acts as a molecular rheostat to promote WASH-dependent endosomal protein recycling and is mutated in a human neurodevelopmental disorder. Mol. Cell.

[11] Li M (2002). Deubiquitination of p53 by HAUSP is an important pathway for p53 stabilization. Nature.

[12] Bhattacharya S, Ghosh MK (2014). HAUSP, a novel deubiquitinase for Rb—MDM2 the critical regulator. FEBS J..

[13] Novellasdemunt L (2017). USP7 is a tumor-specific WNT activator for APC-mutated colorectal cancer by mediating beta-catenin deubiquitination. Cell Rep..

[14] Tavana O (2016). HAUSP deubiquitinates and stabilizes N-Myc in neuroblastoma. Nat. Med..

[15] Song MS (2008). The deubiquitinylation and localization of PTEN are regulated by a HAUSP-PML network. Nature.

[16] Jin Q (2019). USP7 Cooperates with NOTCH1 to drive the oncogenic transcriptional program in T-cell leukemia. Clin. Cancer Res..

[17] Chauhan D (2012). A small molecule inhibitor of ubiquitin-specific protease-7 induces apoptosis in multiple myeloma cells and overcomes bortezomib resistance. Cancer Cell.

[18] Carra G (2017). Therapeutic inhibition of USP7-PTEN network in chronic lymphocytic leukemia: A strategy to overcome TP53 mutated/deleted clones. Oncotarget.

[19] Zhang C (2016). USP7 promotes cell proliferation through the stabilization of Ki-67 protein in non-small cell lung cancer cells. Int. J. Biochem. Cell Biol..

[20] Stolte B (2018). Genome-scale CRISPR-Cas9 screen identifies druggable dependencies in TP53 wild-type Ewing sarcoma. J. Exp. Med..

[21] Fan YH (2013). USP7 inhibitor P22077 inhibits neuroblastoma growth via inducing p53-mediated apoptosis. Cell Death Dis..

[22] Colland F (2009). Small-molecule inhibitor of USP7/HAUSP ubiquitin protease stabilizes and activates p53 in cells. Mol. Cancer Ther..

[23] Kategaya L (2017). USP7 small-molecule inhibitors interfere with ubiquitin binding. Nature.

[24] Hsu HL, Wadman I, Baer R (1994). Formation of in vivo complexes between the TAL1 and E2A polypeptides of leukemic T cells. Proc. Natl. Acad. Sci U.S.A..

[25] O'Neil J, Billa M, Oikemus S, Kelliher M (2001). The DNA binding activity of TAL-1 is not required to induce leukemia/lymphoma in mice. Oncogene.

[26] Nielsen AL, Norby PL, Pedersen FS, Jorgensen P (1996). E-box sequence and context-dependent TAL1/SCL modulation of basic helix–loop–helix protein-mediated transcriptional activation. J. Biol. Chem..

[27] Connelly JP, Pruett-Miller SM (2019). CRISpy: A versatile and high-throughput analysis program for CRISPR-based genome editing. Sci. Rep..

[28] Pagala VR (2015). Quantitative protein analysis by mass spectrometry. Methods Mol. Biol..

[29] Dunham WH, Mullin M, Gingras AC (2012). Affinity-purification coupled to mass spectrometry: Basic principles and strategies. Proteomics.

[30] Zhang Y, Fonslow BR, Shan B, Baek MC, Yates JR (2013). Protein analysis by shotgun/bottom-up proteomics. Chem. Rev..

[31] Downing JR (2012). The Pediatric Cancer Genome Project. Nat. Genet..

[32] Anders S, Pyl PT, Huber W (2015). HTSeq–a Python framework to work with high-throughput sequencing data. Bioinformatics.

[33] Coustan-Smith E (2009). Early T-cell precursor leukaemia: A subtype of very high-risk acute lymphoblastic leukaemia. Lancet Oncol..

[34] Subramanian A (2005). Gene set enrichment analysis: A knowledge-based approach for interpreting genome-wide expression profiles. Proc. Natl. Acad. Sci. U.S.A..

[35] Du X (2018). Hippo/Mst signalling couples metabolic state and immune function of CD8alpha(+) dendritic cells. Nature.

[36] Khatamian A, Paull EO, Califano A, Yu J (2019). SJARACNe: A scalable software tool for gene network reverse engineering from big data. Bioinformatics.

[37] Szklarczyk D (2017). The STRING database in 2017: Quality-controlled protein–protein association networks, made broadly accessible. Nucleic Acids Res..

[38] Ritchie ME (2015). limma powers differential expression analyses for RNA-sequencing and microarray studies. Nucleic Acids Res..

[39] Kharchenko PV, Tolstorukov MY, Park PJ (2008). Design and analysis of ChIP-seq experiments for DNA-binding proteins. Nat. Biotechnol..

[40] Zhang Y, Shin H, Song JS, Lei Y, Liu XS (2008). Identifying positioned nucleosomes with epigenetic marks in human from ChIP-Seq. BMC Genomics.

[41] Ramirez F (2016). deepTools2: A next generation web server for deep-sequencing data analysis. Nucleic Acids Res..

[42] Yang X (2019). Differentiation of human pluripotent stem cells into neurons or cortical organoids requires transcriptional co-regulation by UTX and 53BP1. Nat. Neurosci..

[43] Eng JK, McCormack AL, Yates JR (1994). An approach to correlate tandem mass spectral data of peptides with amino acid sequences in a protein database. J. Am. Soc. Mass Spectrom..

[44] Peng J, Elias JE, Thoreen CC, Licklider LJ, Gygi SP (2003). Evaluation of multidimensional chromatography coupled with tandem mass spectrometry (LC/LC–MS/MS) for large-scale protein analysis: The yeast proteome. J. Proteome Res..

[45] Bai B (2013). U1 small nuclear ribonucleoprotein complex and RNA splicing alterations in Alzheimer's disease. Proc. Natl. Acad. Sci. U.S.A..

[46] Sanda T (2012). Core transcriptional regulatory circuit controlled by the TAL1 complex in human T cell acute lymphoblastic leukemia. Cancer Cell.

[47] Tan TK, Zhang C, Sanda T (2019). Oncogenic transcriptional program driven by TAL1 in T-cell acute lymphoblastic leukemia. Int. J. Hematol..

[48] Cai J (2018). USP7-TRIM27 axis negatively modulates antiviral type I IFN signaling. FASEB J..

[49] Ma X (2018). Pan-cancer genome and transcriptome analyses of 1,699 paediatric leukaemias and solid tumours. Nature.

[50] Wojciechowski J, Lai A, Kondo M, Zhuang Y (2007). E2A and HEB are required to block thymocyte proliferation prior to pre-TCR expression. J. Immunol..

[51] O'Neil J, Shank J, Cusson N, Murre C, Kelliher M (2004). TAL1/SCL induces leukemia by inhibiting the transcriptional activity of E47/HEB. Cancer Cell.

[52] Pierce NW, Kleiger G, Shan SO, Deshaies RJ (2009). Detection of sequential polyubiquitylation on a millisecond timescale. Nature.

[53] Swatek KN, Komander D (2016). Ubiquitin modifications. Cell Res..

[54] Li M, Brooks CL, Kon N, Gu W (2004). A dynamic role of HAUSP in the p53-Mdm2 pathway. Mol. Cell.

[55] Sanda T, Leong WZ (2017). TAL1 as a master oncogenic transcription factor in T-cell acute lymphoblastic leukemia. Exp. Hematol..

