# Supplementary material for: Integrative network analysis reveals USP7 haploinsufficiency inhibits E-protein activity in pediatric T-lineage acute lymphoblastic leukemia (T-ALL)
Source: Sci Rep. 2021 Mar 4;11:5154. doi: 10.1038/s41598-021-84647-2 (PMC7933146; doi:10.1038/s41598-021-84647-2)
Supplement: Supplementary file 1 — Supplementary Figures. [file 41598_2021_84647_MOESM1_ESM.pdf]

Supplementary Materials for:

Title: Integrative Network Analysis Reveals USP7 Haploinsufficiency Inhibits E-protein Activity in Pediatric T-lineage Acute Lymphoblastic Leukemia (T-ALL)

Authors: Timothy I. Shaw[1*], Li Dong[1*], Liqing Tian[1], Chenxi Qian[1], Yu Liu[1], Bensheng Ju[1], Anthony High[2], Kanisha Kavdia[2], Vishwajeeth R. Pagala[2], Bridget Shaner[1], Deqing Pei[3], John Easton[1], Laura J. Janke[4], Shaina N. Porter[5], Xiaotu Ma[1], Cheng Cheng[3], Shondra M Pruett-Miller[5], John Choi[4], Jiyang Yu[1], Junmin Peng[2,7], Wei Gu[8], A. Thomas Look[6], James R Downing[4] & Jinghui Zhang[1§]

Affiliations:

1. Department of Computational Biology, St Jude Children's Research Hospital
2. Center for Proteomics and Metabolomics, St Jude Children's Research Hospital
3. Department of Biostatistics, St Jude Children's Research Hospital
4. Department of Pathology, St Jude Children's Research Hospital
5. Department of Cell and Molecular Biology, St Jude Children's Research Hospital
6. Department of Pediatric Oncology, Dana-Farber Cancer Institute, Harvard Medical School, Boston, MA, 02216, USA
7. Departments of Structural Biology and Developmental Neurobiology, St Jude Children's Research Hospital
8. Department of Pathology and Cell Biology and Institute for Cancer Genetics, Columbia University

§, corresponding author

*, contribute equally

This pdf contains:

Figure S1 – **Additional T-ALL patient analysis**.

Figure S2 – **USP7 shRNA knock down in Jurkat and HSB2 cells.**

Figure S3 – **CRISPR KO in Jurkat cells.**

Figure S4 – **CRISPR KO in Molt-4 cells.**

Figure S5 – **RT-qPCR analysis in Jurkat and Molt-4 USP7 CRISPR KO clones.**

Figure S6 – **De-ubiquitinylation of E2A by USP7 overexpression in HEK293T cells.**

Figure S7 – **Raw western blot images of USP7 in Jurkat and HSB-2 cell lines transduced with USP7 shRNA**

Figure S8 – **Raw western blot images of E-protein after USP7 and TAL1-IP in Jurkat cells.**

Figure S9 – **Raw western blot images of USP7, TRIM27, and ACTIN in Jurkat Cells with CRISPR USP7 KO.**

Figure S10 – **Raw western blot images of E2A, HEB, and ACTIN in Jurkat Cells with CRISPR USP7 KO.**

Figure S11 – **Raw western blot images of USP7, E2A, HEB, TRIM27, and ACTIN in Molt-4 cells with CRISPR USP7 KO.**

Supplementary Figure S1. Additional T-ALL patient analysis.

A

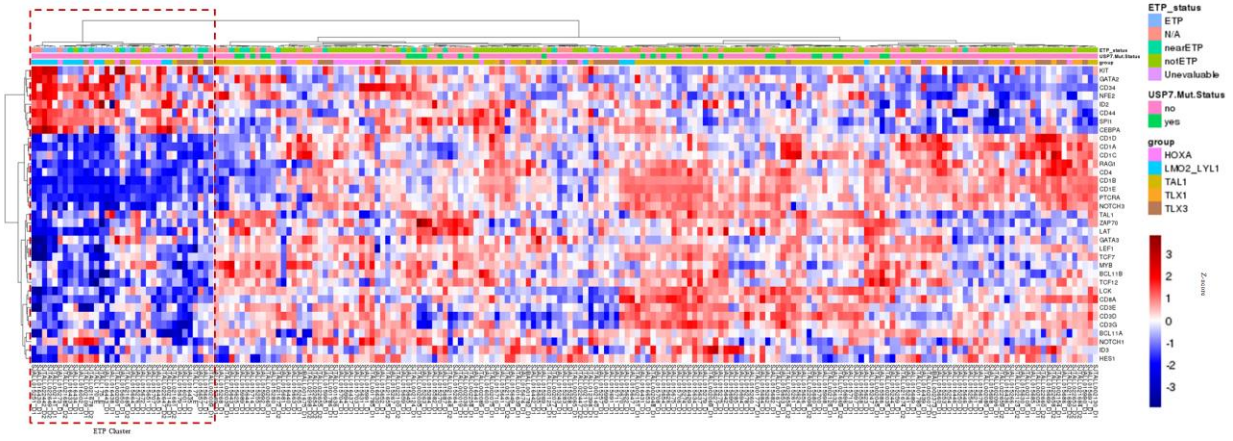

B

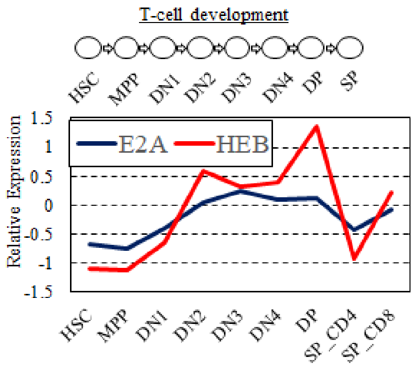

Supplementary Figure S1. Additional T-ALL patient analysis. **A)** Hierarchical clustering reveals early T-cell precursor samples. Genes defining early T-cell precursor leukemias were derived from Coustan-Smith et al. (28). The WARD algorithm was used to perform hierarchical clustering. Known immunohistochemistry annotations supported the ETP cluster definition. **B)** E2A and HEB expression during normal T-cell development.

Supplementary Figure S2. USP7 shRNA knock down in Jurkat and HSB2 cells

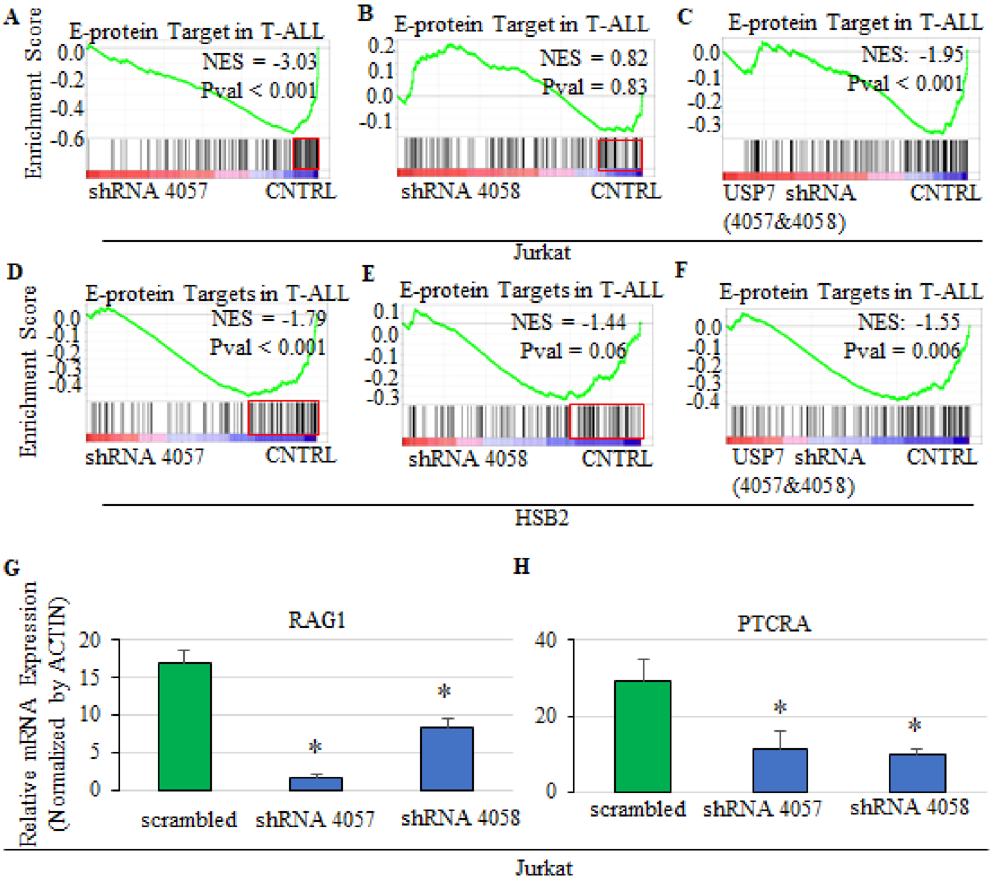

Supplementary Figure S2. Additional analysis of USP7 shRNA knock down in T-ALL cells. The number of RNA-seq samples in each analysis is shown in *Supplementary Table 10*. Enrichment of E-protein targets in USP7^{wt} compared to USP7^{shRNA_4057}, USP7^{shRNA_4058} and combined data set by GSEA analysis in Jurkat cells are shown in **(A)**, **(B)**, and **(C)**, respectively. Leading-edge genes (i.e. significantly down-regulated genes) in individual shRNA knock-downs are marked by a dotted red rectangle, which showed significant overlap (56% gene overlap; Fisher's exact test p < 0.00001). Enrichment of E-protein targets in USP7^{wt} compared to USP7^{shRNA_4057}, USP7^{shRNA_4058}, and the combined data set by GSEA analysis in HSB-2 cells are shown in **(D)**, **(E)** and **(F)**, respectively. Leading-edge genes in individual shRNA knock-downs are marked by a dotted red rectangle, which showed significant overlap (79% gene overlap; Fisher's exact test P < 0.0001). Validation of downregulation of E-protein targets RAG1 **(G)** and PTCRA **(H)** by measuring their relative mRNA quantification normalized by ACTIN in Jurkat. Unpaired two-sided T-test * p-value < 0.05.

Supplementary Figure S3. CRISPR KO in Jurkat cells

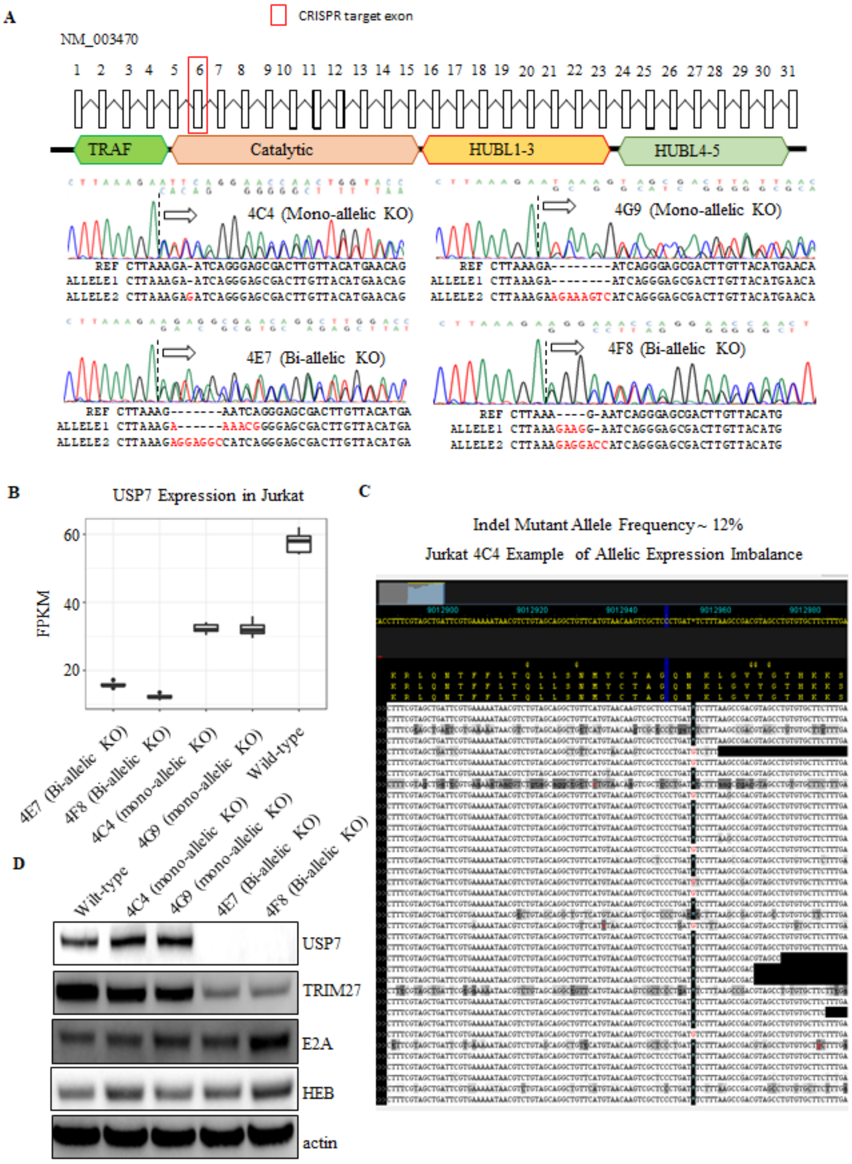

Supplementary Figure S3. **CRISPR KO in Jurkat cells. A)** CRISPR gRNA design targeting USP7's exon six. Sanger validation of the Jurkat CRISPR mono-allelic and bi-allelic USP7 KO models. Red text shows the difference between the mutant allele and the reference allele. B**)** *USP7* RNA expression was reduced by half in mono-allelic KO clones compared to the wild-type. RNA transcript abundance was estimated based on FPKM of RNA-seq. **C)** Reduced expression *USP7* mutant allele in RNA-seq in the 4C4 mono-allelic KO clone. The mutant allele was expressed at 0.12 variant allele fraction in RNA-seq, much lower than the expected 0.50 VAF in DNA, suggesting NMD of the mutant transcript. RNA-seq reads include those of the wild-type allele and those harboring the 1-bp insertion of G (shown in red color) introduced by CRISPR. D) Western blot analysis of USP7, TRIM27, E2A, HEB, and ACTIN in from the Jurkat cells.

Supplementary Figure S4. CRISPR KO in Molt-4 cells

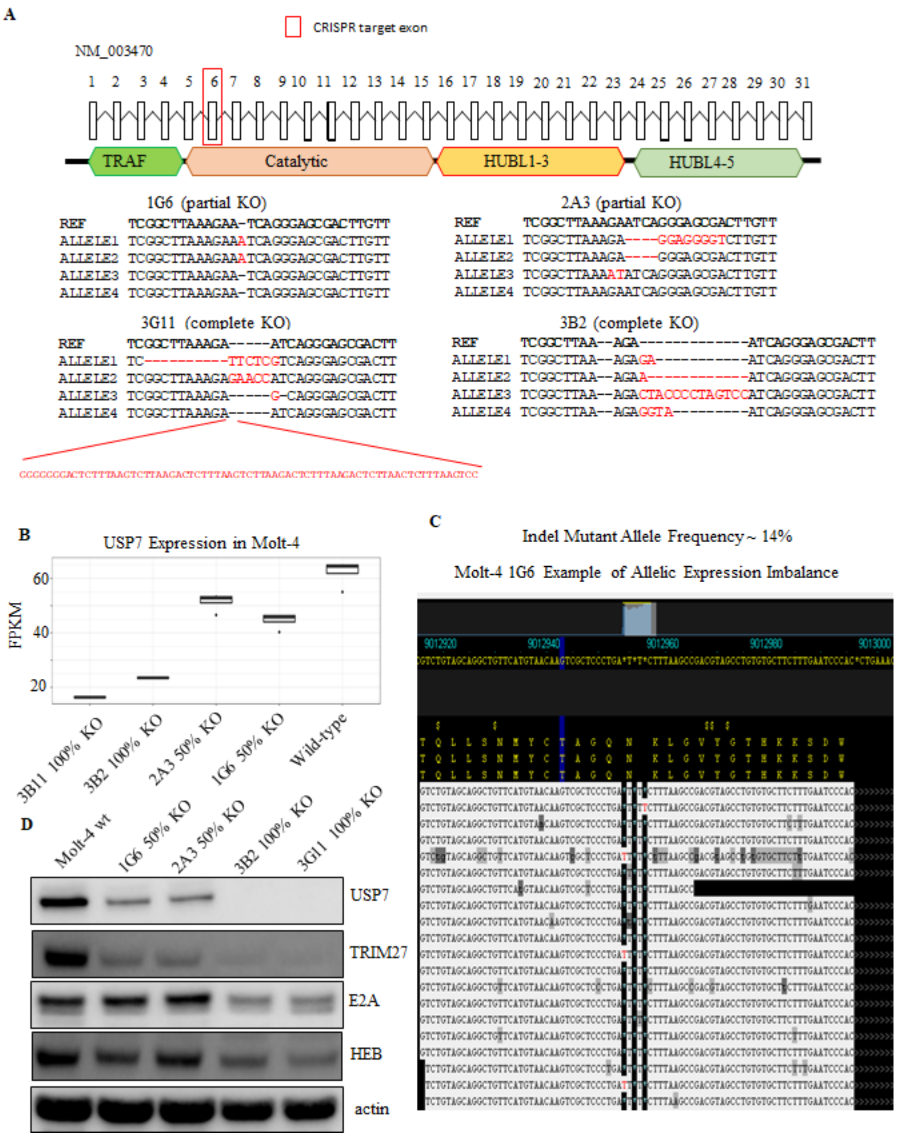

Supplementary Figure S4. CRISPR KO in Molt-4 cells. A) Molt-4 contains four copies of *USP7* and 50% (comparable to haploinsufficiency) and 100% USP7 KO clones were confirmed by MiSEQ sequencing. The red texts highlight the difference between the mutant allele and the reference allele. B**)** *USP7* RNA expression was reduced significantly in 50% KO clones compared to the wild-type. RNA transcript abundance was estimated based on FPKM of RNA-seq. **C)** Reduced expression *USP7* mutant allele in RNA-seq in the 1G6 50% KO clone. The mutant allele was expressed at 0.14 variant allele fraction in RNA-seq, much lower than the expected 0.50 VAF in DNA, suggesting NMD of the mutant transcript. RNA-seq reads include those of the wild-type allele and those harboring the 1-bp insertion of T (shown in red color) introduced by CRISPR. **D)** Western blot analysis of USP7, TRIM27, E2A, HEB, and ACTIN from the Jurkat cell line

Supplementary Figure S5. RT-qPCR analysis in Jurkat and Molt-4 USP7 CRISPR KO clones.

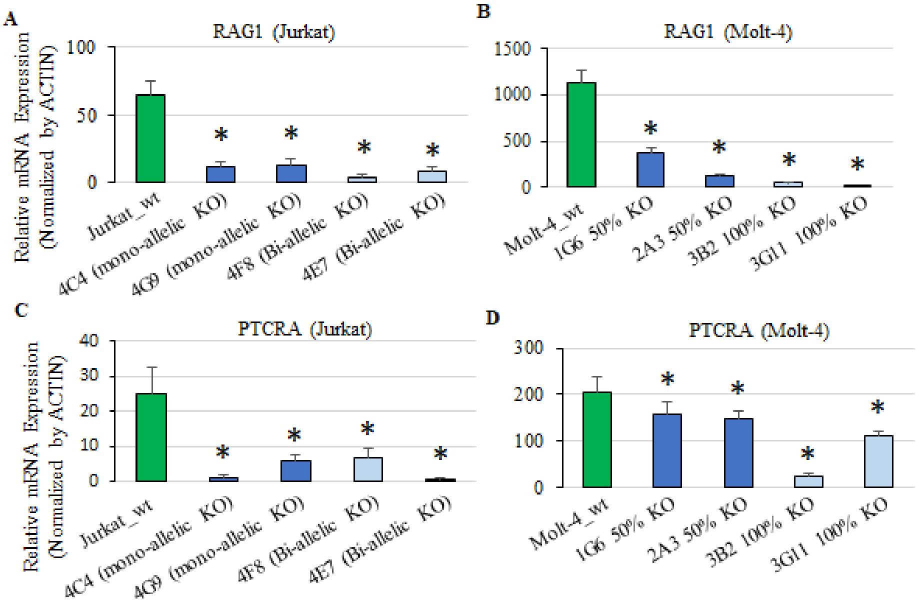

Supplementary Figure S5. RT-qPCR analysis in Jurkat and Molt-4 *USP7* CRISPR KO clones. Relative mRNA quantification by RT-qPCR are normalized by actin in each cell line. *RAG1* relative mRNA quantification is shown in Jurkat (**A**) and Molt-4 (**B**). *PTCRA* relative mRNA quantification is shown in Jurkat (**C**) and Molt-4 (**D**). Unpaired two-sided T-test * p-value < 0.05.

Supplementary Figure S6. De-ubiquitinylation of E2A by USP7 overexpression in HEK293T cells.

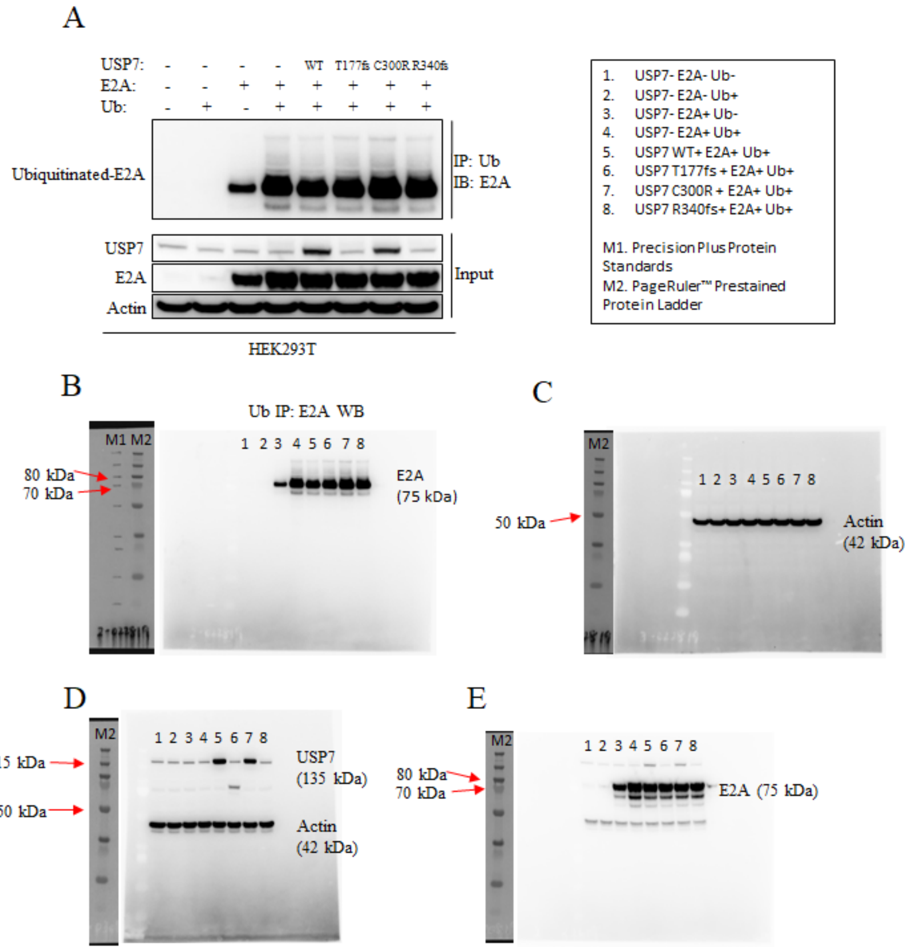

Supplementary Figure S6. De-ubiquitinylation of E2A by USP7 overexpression in HEK293T cells. A) Expression vectors of E2A and ubiquitin were co-transfected with USP7 (WT and mutants) with X-tremeGENE HP DNA transfection reagent (Roche, Indianapolis, IN) for 48 hours (plasmids listed in the Supplementary Table S14). USP7 mutants T177fs, C300R, and R340fs identified in TALL patients were generated by site-direct mutagenesis (Agilent, Santa Clara, CA) on a wild-type USP7 cDNA construct (Addgene Plasmid pCl-neo-Flag-HAUSP from Dr. Bert Vogelstein's lab). Primers used for mutagenesis were listed in Supplementary Table S13. All constructs were sequenced for verification. Proteins were extracted in IP lysis buffer (Thermo Fisher Scientific) and precipitated with anti-ubiquitin antibody (Santa Cruz). Ubiquitinated proteins were separated in NuPAGE 4-12% Bis-Tris protein gels and detected with E2A antibody (Cell Signaling Tech). Expressions of USP7 wild type and mutants were detected in whole cell lysates. Antibodies were listed in the Supplementary Table S12. B) Raw image of Ub IP: E2A WB, ubiquitinated E2A were detected mainly around size 75 kDa with smear tails. C) Raw image of western blotting of actin in whole cell lysates. D) Raw image of western blotting of USP7 in whole cell lysates. E) Raw image of western blotting of E2A in whole cell lysates. Colorimetric images of protein markers M1 or M2 were taken on the same blots.

Supplementary Figure S7. Raw western blot images of USP7 in Jurkat and HSB-2 cell lines transduced with USP7 shRNA (Correspond to the western blot in Figure 3B, 3C).

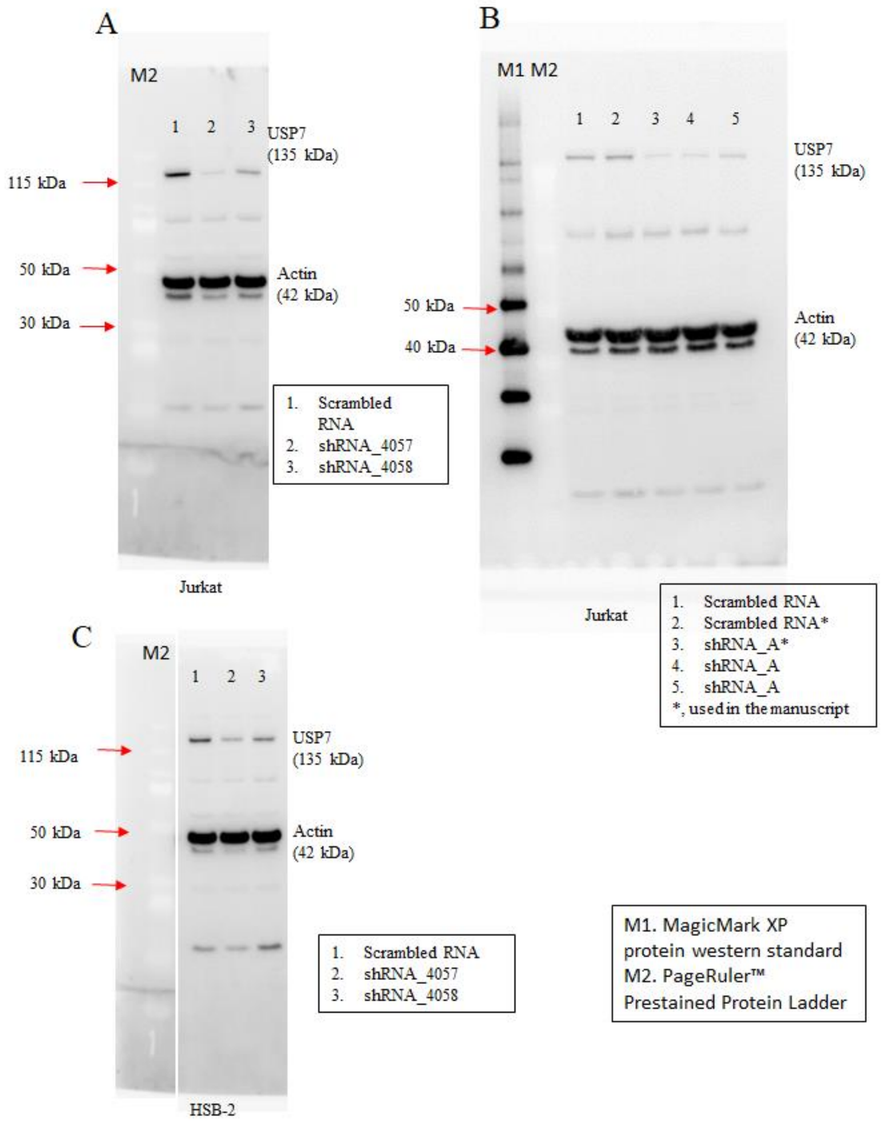

Supplementary Figure S7. Raw western blot images of USP7 in Jurkat (A&B) **and HSB-2** (C) **cell lines transduced with USP7 shRNA.** Antibodies of USP7 and actin used in these blots were listed in supplementary table S12. PageRuler™ Prestained Protein Ladder was used in blots A, B and, C. Protein ladder MagicMark XP protein western standard was used in blot B. All ladders were run in the same blot of the relative images. Predicted protein band sizes were indicated in the blots. **(Correspond to the western blot in Figure 3B, 3C)**

Supplementary Figure S8. Raw western blot images of E-protein after USP7 and TAL1-IP in Jurkat cells.

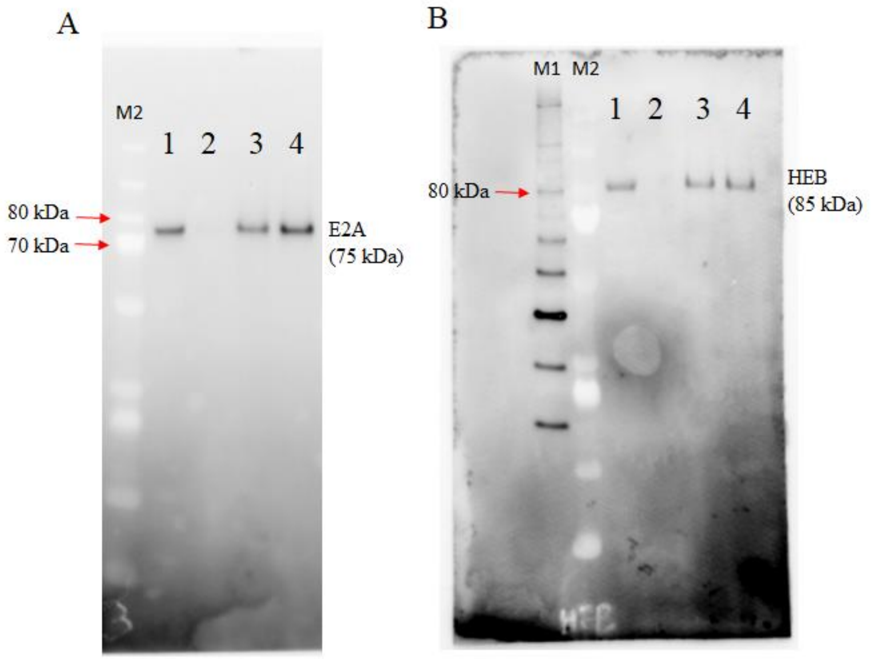

1. Input
2. Ctrl IgG
3. USP7 IP
4. TAL1 IP

M1-MagicMark XP protein western standard
M2-PageRuler™ Prestained Protein Ladder

Supplementary Figure S8. Raw western blot images of E-protein after USP7 and TAL1-IP in Jurkat cells. The same IP samples were used for blotting of E2A (A) and HEB (B). Agarose beads-conjugated mouse anti-USP7 or TAL1 were used for immunoprecipitation, followed by western blotting of rabbit anti-E2A or HEB. Antibodies used in these blots were listed in supplementary table S12. PageRuler™ Prestained Protein Ladder (M2) was run in blots A and B. Additional MagicMark XP protein western standard (M1) was run in blot B. (Corresponds to Figure 4E)

Supplementary Figure S9. Raw western blot images of USP7, TRIM27, and ACTIN in Jurkat Cells with CRISPR USP7 KO (corresponds to Supplementary Figure S3D).

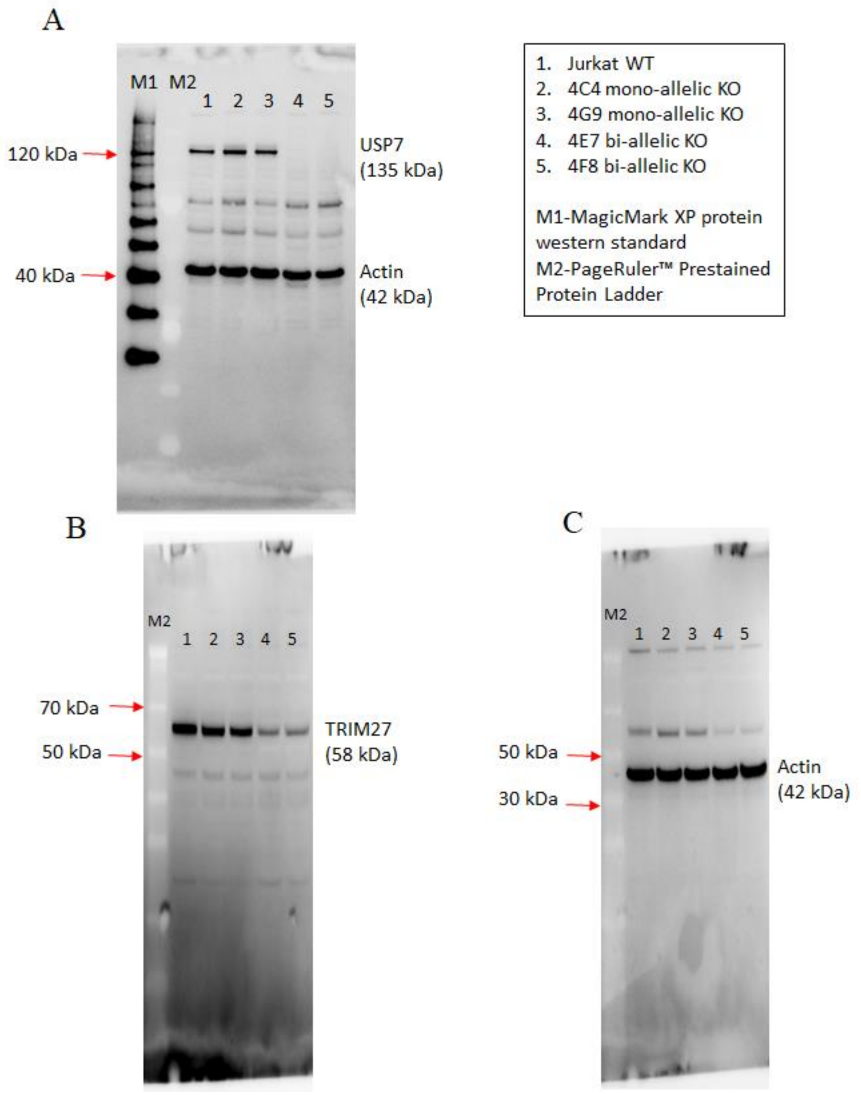

Supplementary Figure S9. Raw western blot images of USP7 and TRIM27 expression in Jurkat CRISPR USP7 KO clones (lanes 1-5 are wild type, clones 4C4, 4G9, 4E7, and 4F8, respectively). USP7, TRIM27, and ACTIN were detected using antibodies list in the supplementary table S12 and labeled on the images with the predicted band size. Protein ladders MagicMark XP protein western standard (M1) and PageRuler™ Prestained Protein Ladder (M2) were run in the same blot of the relative images. The same amount of each sample was loaded in all blots. B and C were developed on the same blot. **(corresponds to Supplementary Figure S3D)**

Supplementary Figure S10. Raw western blot images of E2A, HEB, and ACTIN in Jurkat Cells with CRISPR USP7 KO (Corresponds to Supplementary Figure S3D).

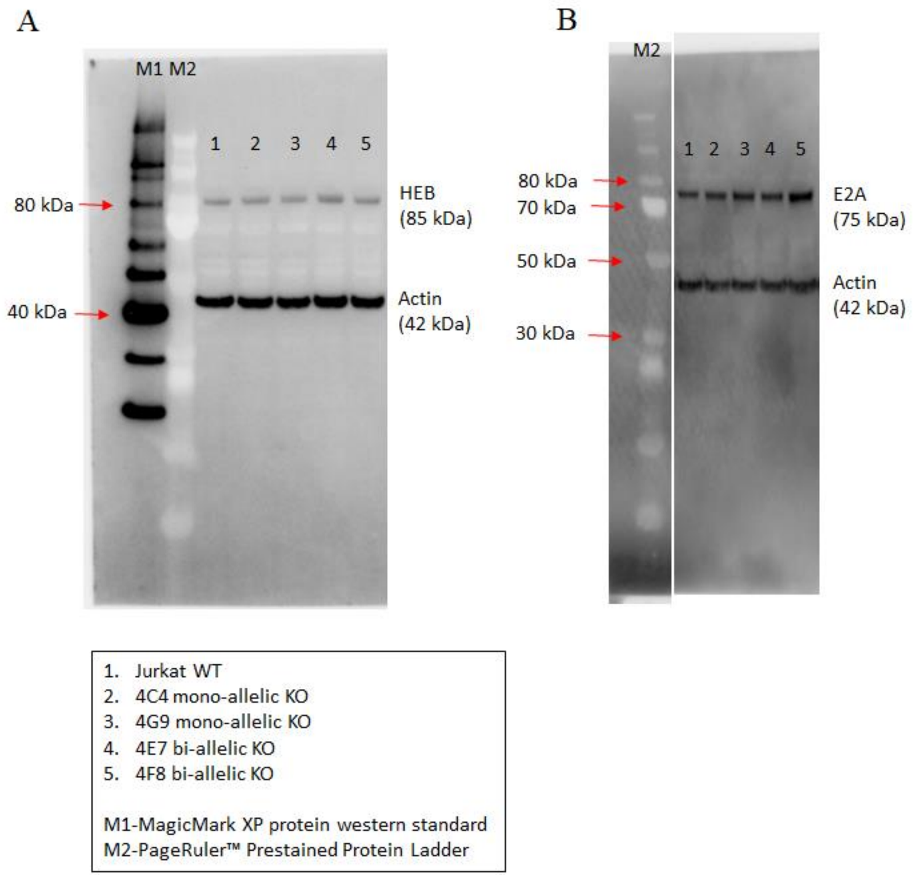

Supplementary Figure S10. Raw western blot images of HEB and E2A expression in Jurkat CRISPR USP7 KO clones (lanes 1-5 are wild type, clones 4C4, 4G9, 4E7, and 4F8, respectively). HEB (A), E2A (B), and actin were detected using antibodies list in the supplementary table S12 and labeled on the images with the predicted band size. Protein ladders MagicMark XP protein western standard (M1) and PageRuler™ Prestained Protein Ladder (M2) were run in the same blot of the relative images. The same amount of each sample was loaded in all blots. **(Corresponds to Supplementary Figure S3D)**

Supplementary Figure S11. Raw western blot images of USP7, E2A, HEB, TRIM27, and ACTIN in Molt-4 cells with CRISPR USP7 KO (Corresponds to Supplementary Figure S4D).

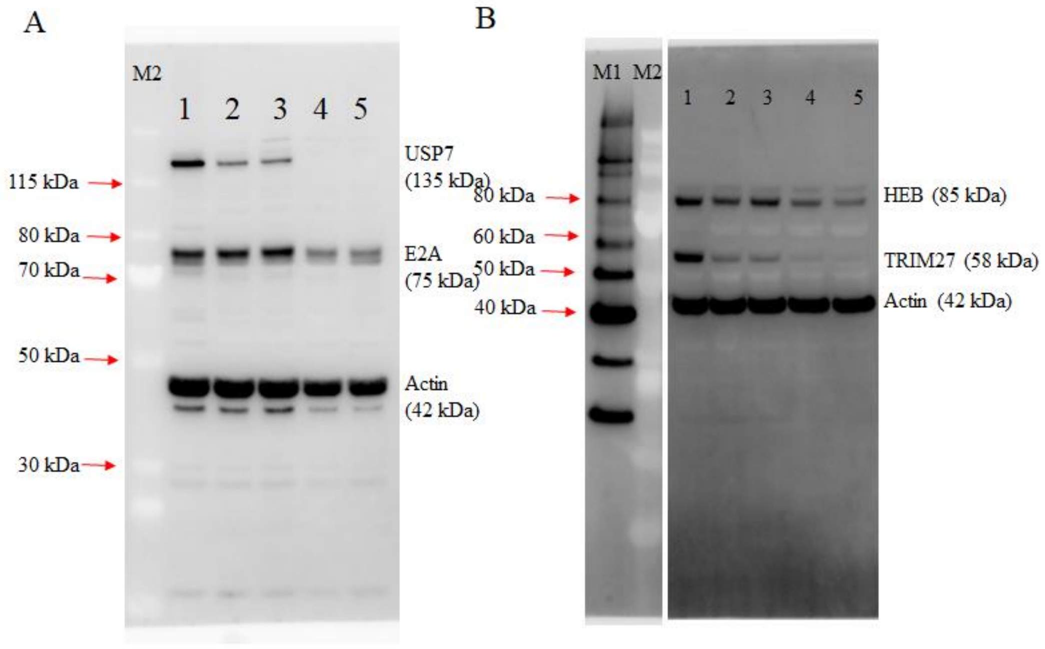

1. Molt-4 WT
2. 1G6 mono KO
3. 2A3 50% KO
4. 3B2 100% KO
5. 3G11 100% KO

M1-MagicMark XP protein western standard
M2-PageRuler™ Prestained Protein Ladder

Supplementary Figure S11. Raw western blot images of USP7, TRIM27, E2A, and HEB in Molt-4 CRISPR USP7 KO clones (lanes 1-5 are wild type, clones 1G6, 2A3, 3B2, and 3G11, respectively). USP7, E2A, and ACTIN (A); HEB and TRIM27 (B) were detected using antibodies listed in the supplementary table S12 and labeled on the images with the predicted band size. Protein ladders MagicMark XP protein western standard (M1) and PageRuler™ Prestained Protein Ladder (M2) were run on the same blot of the relative images. The same amount of each sample was loaded in all blots. **(Corresponds to Supplementary Figure S4D).**
